# Increased Hippocampal ProBDNF Contributes to Memory Impairments in Aged Mice

**DOI:** 10.3389/fnagi.2017.00284

**Published:** 2017-08-31

**Authors:** Mona Buhusi, Chris Etheredge, Ann-Charlotte Granholm, Catalin V. Buhusi

**Affiliations:** ^1^Interdisciplinary Program in Neuroscience, Department of Psychology, Utah State University Logan, UT, United States; ^2^Department of Neuroscience, Medical University of South Carolina Charleston, SC, United States

**Keywords:** BDNF, proBDNF, p75NTR, TAT-Pep5, memory, cofilin, water radial arm maze

## Abstract

Memory decline during aging or accompanying neurodegenerative diseases, represents a major health problem. Neurotrophins have long been considered relevant to the mechanisms of aging-associated cognitive decline and neurodegeneration. Mature Brain-Derived Neurotrophic Factor (BDNF) and its precursor (proBDNF) can both be secreted in response to neuronal activity and exert opposing effects on neuronal physiology and plasticity. In this study, biochemical analyses revealed that increased levels of proBDNF are present in the aged mouse hippocampus relative to young and that the level of hippocampal proBDNF inversely correlates with the ability to perform in a spatial memory task, the water radial arm maze (WRAM). To ascertain the role of increased proBDNF levels on hippocampal function and memory we performed infusions of proBDNF into the CA1 region of the dorsal hippocampus in male mice trained in the WRAM paradigm: In well-performing aged mice, intra-hippocampal proBDNF infusions resulted in a progressive and significant impairment of memory performance. This impairment was associated with increased p-cofilin levels, an important regulator of dendritic spines and synapse physiology. On the other hand, in poor performers, intra-hippocampal infusions of TAT-Pep5, a peptide which blocks the interaction between the p75 Neurotrophin Receptor (p75NTR) and RhoGDI, significantly improved learning and memory, while saline infusions had no effect. Our results support a role for proBDNF and its receptor p75NTR in aging-related memory impairments.

## Introduction

Deficits in learning and memory are frequent in aged individuals (20%–27% among individuals aged 60 and over; Hanninen et al., 1996; Ritchie et al., 2001; Busse et al., 2003; Schönknecht et al., 2005), and a cardinal symptom of dementia. Deficits in memory tasks are related to dysfunctions of the hippocampus (Kadar et al., 1994; West et al., 1994; Rosenzweig and Barnes, 2003), cortex and cholinergic neurons projecting to these areas (Terry and Buccafusco, 2003; McKinney and Jacksonville, 2005; Schliebs and Arendt, 2006). Lesions (Scoville and Milner, 1957; Zola-Morgan et al., 1986; Clark et al., 2000, 2001; Martin et al., 2005), pharmacological or genetic inactivation (Morris et al., 1986; Tsien et al., 1996; Nakazawa et al., 2002, 2003; McHugh et al., 2007), electrophysiological recording (Berger et al., 1983; Pastalkova et al., 2006), molecular imaging (Guzowski et al., 2001; Jones et al., 2001) or MRI imaging studies (Gabrieli et al., 1997; Henke et al., 1997) suggest essential roles for the hippocampus formation in memory. The CA1 region of the hippocampus, the entorhinal cortex and the direct entorhinal-CA1 input are involved in spatial learning and recollection-based spatial memory (Brun et al., 2002, 2008; Moser et al., 2008; Henriksen et al., 2010; Danielson et al., 2016; Igarashi, 2016). CA3 neurons and the CA3-CA1 connection are involved in episodic memory (Nakazawa et al., 2003; Li and Chao, 2008), associative memory recall (Nakazawa et al., 2002) and pattern completion (Gold and Kesner, 2005; Neunuebel and Knierim, 2014). The dentate gyrus is involved in pattern separation (McNaughton and Morris, 1987; Gilbert et al., 2001; Berron et al., 2016; Kesner et al., 2016). Other brain regions thought to be important for memory tasks, particularly for spatial learning and memory, include the anterior thalamus (Warburton et al., 2000; Jankowski et al., 2013), the parietal cortex (Save and Poucet, 2009; Ackerman and Courtney, 2012; Bonnì et al., 2014) and the prefrontal cortex (Rossato et al., 2015; Sapiurka et al., 2016).

*Neurotrophins* are a family of secreted trophic factors that regulate critically important processes in developing and adult brains, including synaptogenesis, synaptic plasticity and neuronal survival (Chao, 2003; Reichardt, 2006). *Brain-Derived Neurotrophic Factor* (BDNF) has emerged as one of the most important molecules for learning and memory (Lu et al., 2014; Hempstead, 2015). Neurotrophins are produced as precursors, which are transferred to secretory vesicles and subjected to either intracellular (Mowla et al., 1999, 2001), or extracellular processing (Smith et al., 1995; Pang et al., 2004) to generate the mature form (Reichardt, 2006; Greenberg et al., 2009). Importantly, precursor brain-derived neurotrophic factor (proBDNF), plasminogen and the *Tissue Plasminogen Activator* (tPA), an enzyme that cleaves plasminogen to generate plasmin, are co-packaged in dense core vesicles present in dendritic spines (Lochner et al., 2008), and can undergo activity-dependent release to mediate changes in synaptic efficacy (Edelmann et al., 2014). Moreover, by regulating the secretion of tPA (Nagappan et al., 2009), neuronal activity controls the ratio of extracellular proBDNF to mature BDNF, which may be crucial for synapse physiology and function, and for neuronal survival.

Mature BDNF and its precursor, proBDNF, have opposing effects on cellular physiology, in line with the “yin-yang” hypothesis (Lu et al., 2005), and similar to its family member NGF (Iulita et al., 2014). Mature BDNF, upon binding to trkB receptors on neuronal membranes, activates ERK signaling and stimulates synaptogenesis, synapse strengthening and neuronal survival (Reichardt, 2006; Hennigan et al., 2007). BDNF and trkB are critical for structural plasticity (increases in spine density and size) and functional plasticity (LTP) in the hippocampus (Korte et al., 1995; Figurov et al., 1996; Patterson et al., 1996) and have important roles in learning and memory (Thoenen, 1995; McAllister et al., 1999; Lu, 2004; Bramham and Messaoudi, 2005). BDNF expression is increased following hippocampus-dependent cognitive tasks (Kesslak et al., 1998; Hall et al., 2000; Mizuno et al., 2000; Bekinschtein et al., 2014). Reduced BDNF or trkB availability in the hippocampus or amygdala, respectively, impairs learning in spatial, aversive or appetitive conditioning paradigms (Linnarsson et al., 1997; Liu et al., 2004; Heldt et al., 2007). Studies aimed at differentiating the role of BDNF in recall vs. acquisition (Mizuno et al., 2000; Lee et al., 2004) showed that BDNF infusion or trkB activation facilitates, while antibodies or antisense oligonucleotides impair fear memory recall (Lee et al., 2004). BDNF also gates the induction of fear extinction (Barnes and Thomas, 2008; Dincheva et al., 2014; Heldt et al., 2014). Extinction memory reactivation increases BDNF and trkB phosphorylation in dorsal CA1, while BDNF infusion after extinction memory reactivation impedes the recovery of the fear response (Radiske et al., 2015).

Instead, proBDNF binds to the pan-neurotrophin receptor p75 Neurotrophin Receptor (p75NTR; Lee et al., 2001), to promote cell death (Frade et al., 1996; Kuan et al., 1999, 2003), to inhibit neurite outgrowth (Yamashita et al., 1999) by modulating RhoA GTPase activity (Yamashita and Tohyama, 2003), and to determine presynaptic terminal retraction (Nakayama et al., 2000; Yang et al., 2009). ProBDNF and p75NTR negatively regulate synaptic transmission and plasticity (Yang et al., 2014; Kailainathan et al., 2015) and are linked to NMDAR-dependent LTD (Nakayama et al., 2000; Rösch et al., 2005; Woo et al., 2005; Yang et al., 2009; Michaelsen et al., 2010). A recently generated cleavage-resistant proBDNF knockin mouse (Yang et al., 2014) revealed that proBDNF negatively regulates hippocampal dendritic complexity and spine density through p75NTR. Conversely, p75-deficient mice have enlarged cholinergic neurons, enhanced LTP and improved spatial learning (Gallagher et al., 1993; Barrett et al., 2010; Endres and Lessmann, 2012) along with other behavioral deficits (Peterson et al., 1999; Wright et al., 2004; Barnes and Thomas, 2008).

Despite the wealth of data gathered through *in vitro* or *ex-vivo* studies, little is known about proBDNF production across the lifespan, its downstream signaling, and consequences of over- or under-expression of proBDNF on learning and memory processes. We hypothesized that aging is associated with a decrease in proBDNF processing to the mature form, which induces alterations in structural and functional plasticity, with detrimental effects upon learning and memory: Thus, increasing proBDNF levels in well-performing mice by local infusions into the CA1 region of the hippocampus would impair spatial memory recall, while blocking the p75NTR effects on RhoA activity in poorly performing aged mice would rescue spatial memory by improving consolidation and recall.

## Materials and Methods

### Subjects

Aged (22–24 months old, *n* = 15) and young (4 months old, *n* = 21) C57BL/6 male mice served as subjects in Experiment 1. Aged (18 months old, *n* = 36) C57BL/6 male mice served as subjects in Experiment 2; 18 months old mice were chosen in Experiment 2 so that they would be closer to gene expression patterns in the aged subjects from Experiment 1 (reduced enzymes for processing, receptor levels, etc.), yet they would be more resilient when subjected to surgical procedures. Mice were housed in a temperature-controlled room under a 12-h light-dark cycle. This study was carried out in accordance with the recommendations of National Institutes of Health, and the Guide for the Care and Use of Laboratory Animals. The protocol was approved by the Utah State University IACUC committee.

### Outline of Studies

**Figure 1:**
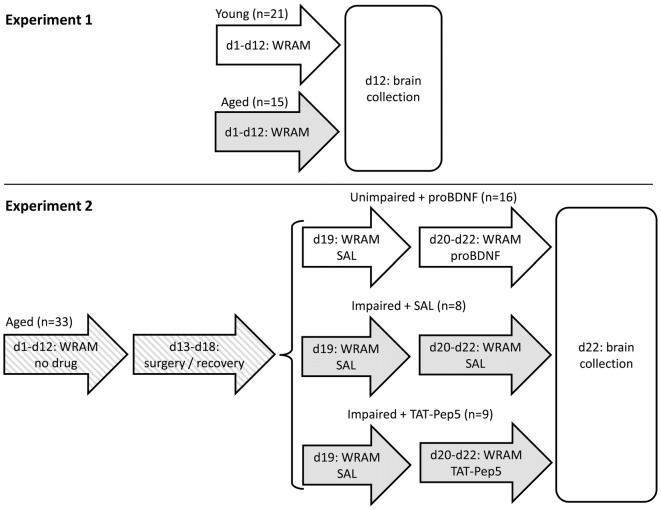
Timeline of Experiments 1 and 2. In Experiment 2, mice were split into three groups based on their performance (unimpaired/impaired) and henceforth infused with different drugs (saline, proBDNF, TAT-Pep5). d, day of study; WRAM, behavioral training/testing in the water radial arm maze task; SAL, saline.

In Experiment 1, aged 22–24 old mice and young 4 months old mice were trained for 12 days (four blocks of three daily sessions) in a water radial arm maze (WRAM), which provides a reliable, sensitive test of learning and memory (reference memory (RM) and working memory (WM) and WM load), combining the advantages of the Morris water maze and radial arm maze (Morgan et al., 2000; Shukitt-Hale et al., 2004; Alamed et al., 2006). Unlike the land version of the RAM, the WRAM does not require food restriction as motivation, which is critical given reports linking food restriction to improved cognitive function and changes of neurotrophin levels (Komatsu et al., 2008; Kumar et al., 2009; Witte et al., 2009). Afterwards, fresh whole hippocampus was collected, and levels of BDNF, proBDNF, p-trk140, trkB140, p75, carboxypeptidase E (CPE) and tPA in hippocampal lysates were quantified in aged and young mice. The timeline of the study is shown in Figure 1.

In Experiment 2 we aimed at further evaluating the role of hippocampal proBDNF in learning and memory in the WRAM task, and the possible role of p75 signaling downstream proneurotrophin in improving performance in aged mice. We have chosen not to infuse these drugs in young mice since results from Experiment 1 indicate young mice have different expression of enzymes and receptors, and may process proBDNF into BDNF using different enzymes. On the other hand, the aged rodent population—in as much as the human aged population—is known to be highly heterogeneous in regard to memory performance (Gallagher et al., 1993): Some aged individuals are memory impaired while others are not, so effects of drug manipulations are generally evaluated in a mixed population of poor and good performers. Moreover, in regard to experimental design, one has to take into account a possible “floor effect” in memory errors (when starting with a good performance, learning can hardly be improved further) and a “ceiling effect” (an animal with poor learning and memory, making numerous errors, can hardly perform much worse). Therefore, rather than injecting all drugs in both poor and good performers, in Experiment 2 good performers (unimpaired aged mice) received proBDNF infusions in the hippocampus, in order to assess its role in decreasing performance, while poor performers (memory-impaired aged mice) were infused with TAT-Pep5 to evaluate the role of p75 signaling downstream proneurotrophin in possibly improving their performance.

More specifically, in Experiment 2, 18-month old aged mice were trained for 12 days in the WRAM task. Based on their average performance on the last block of three daily sessions before surgery (d10–12, see Figure 1 for a time line of the study), mice showing less than four memory errors were assigned to a well-performing (unimpaired) group, to be infused in d20–22 with proBDNF (unimpaired + proBDNF; average WM errors ± SEM = 2.0 ± 0.3, range 0–3; RM errors = 1.6 ± 0.3, range 0–3). The remaining (impaired) mice showed at least four memory errors (WM errors = 7.2 ± 1.8, range 4–17; RM errors = 6.3 ± 1.3, range 4–12) and were further randomly assigned to either a group to be infused in d20–22 with TAT-Pep5 (impaired + TAT-Pep5) or saline control (impaired + SAL). The impaired + SAL group was deemed to be a direct control for the “impaired + TAT-Pep5” group, to control for possible improvement of performance due to repeated testing, to control for possible degradation in performance as a result of potential lesions due to repeated infusion procedure, and to also provide a landmark on performance of aged mice in the WRAM, to be contrasted both against the effect of TAT-Pep5 on performance of impaired mice, and against the effect of proBDNF on performance of unimpaired mice. All mice received surgery for cannula implantation directed at the CA1 region of the dorsal hippocampus, and were allowed to recover for 1 week before being tested in the WRAM task for four more days (days 19–22). On d19 all mice were infused with saline (SAL) to evaluate stable performance pre- and post-surgery. On d20–22 mice were infused as described above. The timeline of the study is shown in Figure 1. Details of procedures are provided below.

### Behavioral Procedures

We used an 8-arm WRAM with four hidden platforms, placed in a room with salient extra-maze cues that remained constant during testing. Each subject was trained/tested in one session per day (four trials to locate the four platforms), for 12 consecutive days (for local infusion studies, subjects received 4 extra testing sessions under drug infusion). The subject was released from the center of the maze and had 3 min to locate a platform. Once a platform was found, the animal remained on it for 15 s and was returned to a heated cage for 30 s; the found platform was removed, and the procedure was repeated until all four platforms were identified. With every trial it becomes increasingly difficult to recall previous entries and locate remaining platforms: 4/8 in trial 1, 3/8 in trial 2, 2/8 in trial 3, 1/8 in trial 4, thus increasing the memory load as trials within each day progressed. All arm entries were recorded. Errors were quantified for each daily session as WM errors (entries in previously visited arms) and RM errors (entries in arms that never contained platforms; Jarrard et al., 1984). The platform set-up was chosen randomly for each mouse at the beginning of testing, then maintained constant throughout the experiment.

### Surgical Procedures

After 12 days of WRAM training, mice were implanted with stainless steel double cannula guides (28GA, PlasticsOne), bilaterally aimed at the dorsal hippocampus, using a stereotactic apparatus under aseptic conditions and anesthesia. Guides were fixed with dental cement and covered with caps. Mice were allowed to recover for 1 week before being retested and locally infused as described below.

### Drug Delivery Regimen

Infusions were performed using a gear-driven infusion pump (Harvard Apparatus, Hollistone, MA, USA) with Hamilton syringes connected to the internal cannulae via polyethylene tubing. Internal double cannulae (33GA) extended 0.5 mm beyond the cannula guides and tips were directed at the CA1 subfield of the dorsal hippocampus (AP −2.1, ML ±1.5, DV −1.5) and (AP −2.7, ML ±2.1, DV −1.5; Franklin and Paxinos, 2008). After recovery from surgery, on d19 all mice received intra-hippocampal infusions of saline, to test stable performance before and after surgery. On d20–22 unimpaired mice in group “unimpaired + proBDNF” were injected with “*uncleavable*” mouse proBDNF (proBDNF mut-mouse, Alomone, Jerusalem, Israel) 40 pg/0.4 μL/side. “Uncleavable” proBDNF differs from wildtype proBDNF at the site of cleavage by plasmin and was used in order to delay *in vivo* processing of proBDNF. On d20–22, underperforming mice in group “impaired + TAT-Pep5” were injected with a solution of TAT-Pep5 (EMD Millipore, Billerica, MA, USA) 4 ng/0.4 μL/side, while underperforming mice in “group impaired + SAL” were injected with saline 0.4 μL/side. All drug solutions were infused at a speed of 0.1 μL/min. Cannulae were left in place an extra 2 min post infusion. Patency of cannulae was tested after each injection. Ten minutes after infusion, mice were tested in the WRAM.

### Histological Analysis of Cannula Placement

After the last day of testing, mice were sacrificed and brains collected for histological analyses to ascertain the placement of cannulae. Mice were deeply anesthetized with isoflurane and transcardially perfused with 4% paraformaldehyde solution. Brains were collected and sectioned at 50 μm thickness on a vibrating microtome (Leica VT1200S, Germany). Sections were placed on positively charged glass slides, rehydrated and stained with a 0.1% cresyl violet solution, then cleared and coverslipped with Permount. Sections were examined for cannula placement on a Zeiss AxioImager M2 motorized research microscope with an imaging system. Only animals with cannulae correctly placed were used for analyses. Two mice in group impaired + SAL and one mouse in group unimpaired + proBDNF were eliminated for improper cannula placement and/or clogged cannula guides.

### Protein Biochemistry

**Figure 2:**
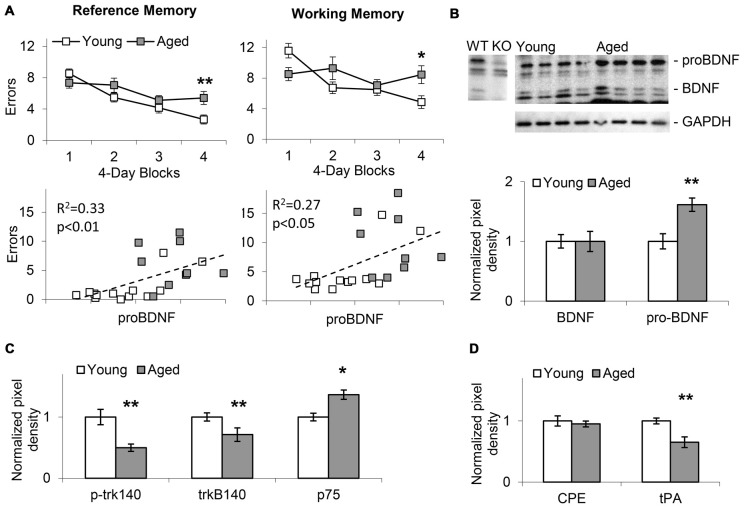
Memory impairment in aged mice positively correlates with increases in hippocampal proBDNF levels. **(A)** Average (±SEM) reference memory (RM) and working memory (WM) errors over four 4-session blocks of a WRAM task in 24-month old aged mice and 4-month old young mice. RM and WM errors positively correlate with proBDNF levels. **(B)** Increase in proBDNF, but not brain-derived neurotrophic factor (BDNF), in 24-month old aged mice relative to young 4-month old mice. **(C)** Relative to young 4-month old mice, aged 24-month old mice show increased p75 Neurotrophin Receptor (p75NTR) and decreased p-trk140 and trk140. **(D)** Relative to young 4-month old mice, aged 24-month old mice show decreased Tissue Plasminogen Activator (tPA), but not carboxypeptidase E (CPE). *p < 0.05; **p < 0.01.

Fresh whole hippocampus was collected and stored in liquid nitrogen (Barnes and Thomas, 2008). Tissue was homogenized in lysis buffer (50 mM Tris, 150 mM NaCl, 1% Triton X-100, 50 mM octylglucoside, 2 mM EDTA, 2 mM EGTA, protease and phosphatase inhibitors). Equal protein amounts (20 μg) were separated onto 4%–20% Novex gels (Invitrogen, Carlsbad, CA, USA) and transferred to PVDF membranes. Proteins were visualized using primary antibodies, HRP-conjugated secondary antibodies (Jackson Immunoresearch, West Grove, PA, USA) and ECL (ThermoFisher Scientific, Grand Island, NY, USA). The following antibodies were used: anti-BDNF, anti-TrkB and anti-pTrk and anti-CPE (Santa Cruz Biotechnology, Dallas, TX, USA), anti-p75NTR (ABCAM, Cambridge, MA, USA), anti-tPA (American Diagnostica, Stamford, CT, USA). Cofilin and phospho-cofilin (Ser3) were obtained from Cell Signaling Technologies (Danvers, MA, USA). Antibodies were validated by the manufacturer and other users and tested for specificity in our laboratory. After evaluating several BDNF antibodies, sc-546 (Santa Cruz Biotechnology, Dallas, TX, USA) was chosen as the only one to reliably identify both BDNF (14 kDa) and proBDNF (approx. 30 kDa; see comparison to BDNF KO brain lysates, Figure 2B). Equal protein loading was verified using an HRP-conjugated anti-GAPDH antibody (ABCAM, Cambridge, MA, USA); major products were quantified (relative to GAPDH and to a standard brain lysate) using a FluorChem9900 system (Alpha Innotech, San Leandro, CA, USA).

### Statistical Analyses

In Experiment 1, WM errors (entries in previously visited arms) and RM errors (entries in arms that never contained platforms; Jarrard et al., 1984) were subjected to mixed ANOVAs with between-subjects variable age (aged, young) and within-subjects variable block (four blocks), followed by *post hoc* analyses. Levels of BDNF, proBDNF, p-trk140, trkB140, p75, CPE and tPA relative to GAPDH in hippocampal lysates were normalized to the average levels found in young mice, and subjected to *Student t* tests.

In Experiment 2, baseline memory performance (WM and RM errors) was evaluated in mixed ANOVAs with between-subjects variable group (three groups) and within-subjects variable session (before surgery: average performance in the last block of three daily sessions before surgery, and after surgery: d19 first session after surgery), followed by *post hoc* analyses. Memory performance (WM and RM errors) in the impaired + SAL group was further evaluated in repeated-measures ANOVAs with within-subjects variable session (four local SAL infusion sessions: d19–d22) followed by *post hoc* analyses. Memory performance (WM and RM errors) in the unimpaired + proBDNF group relative to the impaired + SAL group was evaluated in mixed ANOVAs with between-subjects variable group (two groups) and within-subjects variable session (four drug infusion sessions: d19–d22) followed by *post hoc* analyses. Memory performance (WM and RM errors) in the impaired + TAT-Pep5 group relative to the impaired + SAL group was evaluated in mixed ANOVAs with between-subjects variable group (two groups) and within-subjects variable session (four drug infusion sessions: d19–d22) followed by *post hoc* analyses. Levels of p-cofilin to total cofilin ratio were normalized to the average ratio found in unimpaired mice and subjected to a one-way ANOVA with factor group (three groups), followed by *post hoc* analyses. Statistical analyses were performed in STATISTICA (StatSoft, Palo Alto, CA, USA). All statistical analyses were conducted at an alpha level 0.05.

## Results

### Spatial Reference and Working Memory Errors in the WRAM Increase with Age, and Are Positively Correlated with proBDNF Levels

In Experiment 1, WRAM spatial RM and WM performance was evaluated in aged (22–24-months old, *n* = 15) and young (4-months old, *n* = 21) mice as shown in the upper panels of Figure 2A. Mice showed improvement in WRAM performance (decrease in number of errors) over blocks of four daily sessions both in RM (*F*_(3,102)_ = 16.58, *p* < 0.01) and WM (*F*_(3,102)_ = 5.54, *p* < 0.01). However, analyses also indicated an age × block interaction both in regard to RM (*F*_(3,102)_ = 3.69, *p* < 0.05) and WM (*F*_(3,102)_ = 4.80, *p* < 0.01) suggesting that aged mice showed less improvement over sessions relative to young mice. Indeed, performance was significantly impaired in aged mice relative to young mice in the last block of the WRAM task both in regard to RM (*F*_(1,34)_ = 7.96, *p* < 0.01) and WM (*F*_(1,34)_ = 6.67, *p* < 0.05). The lower panels of Figure 2A indicate the pattern of RM and WM errors relative to proBDNF levels (quantified relative to GAPDH) in aged and young mice. Plots indicate a relative diversity of the pattern at both ages. Most importantly, irrespective of age, proBDNF levels correlated significantly both with RM errors (*R*^2^ = 0.33, *p* < 0.01) and WM errors (*R*^2^ = 0.27, *p* < 0.05).

### Aging Is Characterized by Changes in BDNF Processing and Signaling Pathways

Western blot analyses of hippocampal lysates (Figure 2B) suggested that aged animals exhibit an increase in the proBDNF to BDNF ratio when compared to young mice. Quantification of BDNF and proBDNF relative to GAPDH in 22–24-months old aged mice (*n* = 9) and young 4-month old mice (*n* = 13) indicated a significant increase in proBDNF levels with age (*t*_(20)_ = 5.14, *p* < 0.01), but no significant difference in BDNF levels (*t*_(20)_ < 0.01, *p* > 0.05). Moreover, as indicated in Figure 2C, we identified a significant decrease in p-trk140 (*t*_(20)_ = 5.45, *p* < 0.01), and trkB140 (*t*_(20)_ = 3.26, *p* < 0.01), and a significant increase in p75NTR levels (*t*_(20)_ = 2.42, *p* < 0.05) in aged mice relative to young mice; no changes were found in trkB95 levels (*t*_(20)_ = 1.69, *p* > 0.05). Evaluation of tPA, which activates plasmin, an enzyme required for proBDNF processing (Pang et al., 2004), and CPE, a protein required for both cellular transport and processing of proBDNF (Park et al., 2008), revealed decreased tPA levels (*t*_(20)_ = 5.13, *p* < 0.01), but not CPE levels (*t*_(20)_ = 0.74, *p* > 0.05), in aged mice relative to young (Figure 2D). Overall, these results suggest that proBDNF processing and receptor levels in the hippocampus changed with age, and that manipulating proBDNF levels and receptor activation may alter both RM and WM spatial performance in the WRAM task. This hypothesis was tested by observing the effects of local hippocampal infusions of proBDNF and TAT-Pep5 on performance in the WRAM task in aged mice.

### Stable Memory Performance Pre- and Post-Surgery

**Figure 3:**
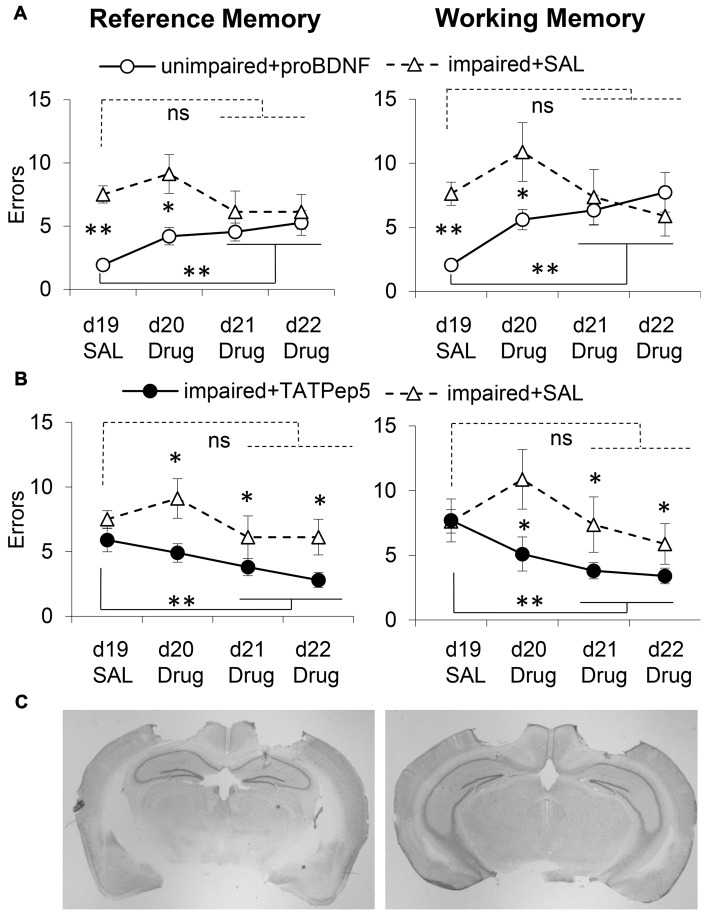
Intra-hippocampal infusion of proBDNF impairs memory in well-performing (unimpaired) mice, while TAT-Pep5 infusion improves memory in poorly-performing (impaired) mice. **(A)** Average (±SEM) RM and WM errors in memory impaired 18-month old mice receiving intra-hippocampal saline infusions (impaired + SAL, *n* = 8, open triangles) and well-performing 18-month old aged mice receiving uncleavable proBDNF intra-hippocampal infusions (unimpaired + proBDNF, *n* = 16, open circles) over four daily sessions of a WRAM task. **(B)** Average (± SEM) RM and WM errors in memory impaired 18-month old mice infused with saline (impaired + SAL, *n* = 8, open triangles) and memory impaired 18-month old mice receiving intra-hippocampal infusions of TAT-Pep5 (impaired + TAT-Pep5, *n* = 9, closed circles) over four daily sessions of a WRAM task. **(C)** Representative images indicating the locations of drug infusions at two levels of the hippocampus. *ns* not significant; *p < 0.05; **p < 0.01.

Analyses of memory performance before and after surgery failed to indicate differences in performance before and after surgery for either RM (*F*_(1,30)_ = 0.48, *p* > 0.05) or WM (*F*_(1,30)_ = 0.22, *p* > 0.05), or any interactions with this variable for either RM (*F*_(2,30)_ = 0.18, *p* > 0.05) or WM (*F*_(2,30)_ = 0.01, *p* > 0.05), indicating stable performance pre- and post-surgery. However, analyses indicated differences between groups for both RM (*F*_(2,30)_ = 30.39, *p* < 0.05) and WM (*F*_(2,30)_ = 27.63, *p* < 0.05). For both RM and WM errors, *post hoc* Tukey tests indicated reliable differences between impaired and unimpaired mice (*p* > 0.05), but no differences between the two groups of impaired mice (*p* > 0.05), both before and after surgery. Figures 3A,B show the average number of RM (left) and WM (right) errors (±SEM) in the WRAM task in days 19–22 in unimpaired + proBDNF mice (*n* = 16, open circles), impaired + TAT-Pep5 mice (*n* = 9, closed circles), and impaired + SAL mice (*n* = 8, open triangles). Figure 3C shows representative images detailing the CA1 infusion location.

### No Improvement in Performance in Memory-Impaired Mice Infused with Saline in the CA1 Region of the Dorsal Hippocampus

WM and RM errors for the memory impaired mice infused with saline (impaired + SAL, *n* = 8, open triangles) are shown in Figure 3A. For both RM and WM errors, analyses failed to indicate an effect of session for either RM (*F*_(3,21)_ = 0.98, *p* > 0.05) or WM (*F*_(3,21)_ = 1.41, *p* > 0.05), suggesting that memory impaired mice receiving intra-hippocampal infusions with saline failed to improve reliably over the four sessions. The performance of memory impaired mice infused with TAT-Pep5, and of unimpaired (well-performing) mice infused with proBDNF was evaluated relative to that of memory impaired mice infused with saline as discussed below.

### Decline in Working and Reference Memory in Unimpaired Mice Infused with proBDNF in the CA1 Region of the Dorsal Hippocampus

On days d20–22 unimpaired mice were infused with proBDNF (unimpaired + proBDNF, *n* = 16, open circles), while memory impaired mice were infused with saline (impaired + SAL, *n* = 8, open triangles). Figure 3A shows behavioral performance in regard to RM (left) and WM (right) in the two groups. Analyses suggest that proBDNF infusions over d20–22 produced a considerable decline in performance relative to d19. Analyses indicated a reliable main effect of group for both RM (*F*_(1,22)_ = 19.22, *p* < 0.05) and WM (*F*_(1,22)_ = 5.44, *p* < 0.05), indicating that over the four sessions unimpaired mice performed better than memory impaired mice. Analyses also indicated a reliable group × session interaction for both RM (*F*_(3,66)_ = 3.46, *p* < 0.05) and WM (*F*_(3,66)_ = 3.09, *p* < 0.05), suggesting that the performance of unimpaired mice infused with proBDNF declined (mice made more errors) with repeated proBDNF infusions. While on d19 unimpaired + proBDNF mice performed reliably better than memory-impaired controls both in RM (*F*_(1,22)_ = 50.03, *p* < 0.01) and WM (*F*_(1,22)_ = 42.83, *p* < 0.01), their performance declined with proBDNF infusions, such that on d21–22 there was no reliable difference from that of memory-impaired controls, neither in RM (*Fs*_(1,22)_ = 1.29, *p* > 0.05) nor in WM (*F*_(1,22)_ = 0.02, *p* > 0.05). Furthermore, following proBDNF infusion, unimpaired + proBDNF mice performed significantly worse during the last 2 days d21–22 than on d19, for both RM (*F*_(1,22)_ = 13.25, *p* < 0.01) and WM (*F*_(1,22)_ = 21.09, *p* < 0.01), suggesting that proBDNF infusion had a considerable worsening effect on both WM and RM performance (Figure 3A).

### Infusion of TAT-Pep5 in the CA1 Region of the Dorsal Hippocampus Improved Working and Reference Memory in Impaired Aged Mice

To assess whether blocking signaling downstream the proBDNF receptor, p75NTR, would have opposite effects on spatial learning and memory, i.e., would improve WRAM performance, we evaluated the effects of infusions of TAT-Pep5, a peptide which interferes with the interaction between p75NTR and Rho-GDI, thus modulating RhoA activity (Yamashita and Tohyama, 2003). Impaired mice were randomly assigned to two groups infused in d20–22 with either TAT-Pep5 (impaired + TAT-Pep5, *n* = 9, closed circles) or saline control (impaired + SAL, *n* = 8, open triangles). Figure 3B shows behavioral performance in regard to RM (left) and WM (right) in the two groups, and indicates that TAT-Pep5 infusions over d20–22 produced a gradual improvement in performance relative to d19. Analyses indicated a reliable main effect of group for both RM (*F*_(1,15)_ = 14.19, *p* < 0.05) and WM (*F*_(1,15)_ = 9.27, *p* < 0.05), indicating that over the 3 sessions memory-impaired mice infused with TAT-Pep5 performed better both in RM and WM than saline controls. Analyses failed to indicate a main effect of session on both RM (*F*_(3,45)_ = 2.46, *p* > 0.05) and WM (*F*_(3,45)_ = 2.19, *p* > 0.05), or group × session interactions for either RM (*F*_(3,45)_ = 0.69, *p* > 0.05) or WM (*F*_(3,45)_ = 1.79, *p* > 0.05). While on d19 performance was not different in the two groups neither for RM (*F*_(1,15)_ = 1.43, *p* > 0.05) or WM (*F*_(1,15)_ = 0.02, *p* > 0.05), over sessions with TAT-Pep5 infusions performance improved such that on d21–22 performance was reliably better than that of control mice infused with saline both in RM (*F*_(1,15)_ = 5.85, *p* < 0.05) and WM (*F*_(1,15)_ = 5.97, *p* < 0.05). Furthermore, following TAT-Pep5 infusion, impaired + TAT-Pep5 mice performed significantly better during last 2 days d21–22 than on d19, for both RM (*F*_(1,15)_ = 7.07, *p* < 0.05) and WM (*F*_(1,15)_ = 7.28, *p* < 0.05), suggesting that TAT-Pep5 infusion had a beneficial effect on both WM and RM performance (Figure 3B).

### Increased p-Cofilin Levels in the Hippocampi of Impaired Mice and of Unimpaired proBDNF-Infused Mice

Neuronal plasticity is essential for learning and memory. Rapid reorganization of the actin skeleton is an important factor for neuronal plasticity (Segal, 2017): for example, the actin filament depolymerizing protein ADF/cofilin controls dendritic spine morphology, as well as synaptic availability of AMPA receptors, and exocytosis of synaptic vesicles (Rust, 2015). Loss of cofilin-mediated synaptic actin dynamics leads to impairment of all types of associative learning (Rust et al., 2010). Since phosphorylation of cofilin is an end target for RhoA pathways (Rex et al., 2009; Briz et al., 2015), we have analyzed p-cofilin levels as a neuronal plasticity marker, in mice showing memory impairments in the WRAM, well-performing mice and well-performing mice receiving proBDNF infusions in the hippocampus.

**Figure 4:**
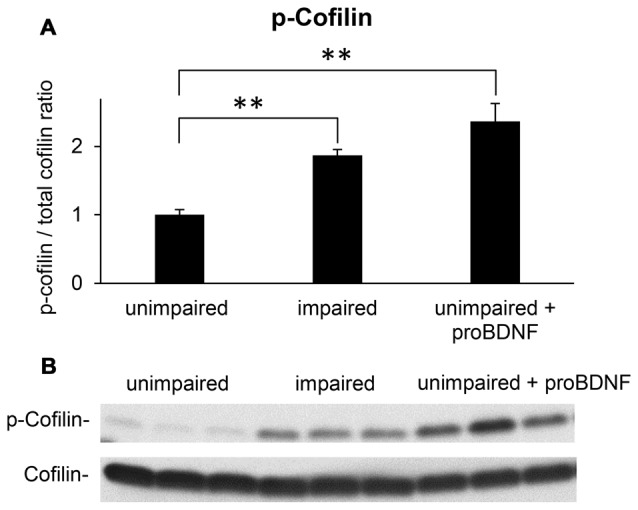
Intra-hippocampal infusion of proBDNF increases p-cofilin levels in memory-unimpaired mice to levels seen in memory-impaired mice. **(A)** p-Cofilin to total cofilin ratio in memory-unimpaired mice, memory-impaired mice, and memory-unimpaired mice infused with proBDNF. **(B)** Representative p-cofilin and cofilin blots. **p < 0.01.

Western blot analyses of hippocampal lysates suggested that mice showing memory impairments in the WRAM exhibited an increase in the p-cofilin to cofilin ratio when compared to well-performing mice (Figure 4B). Quantified levels of p-cofilin to total cofilin ratios in memory impaired mice (*n* = 7), unimpaired mice (*n* = 7), and unimpaired mice infused with proBDNF (*n* = 5) were subjected to a one-way ANOVA, which indicated a main effect of group (*F*_(2,16)_ = 18.42, *p* < 0.01). *Post hoc* comparisons (Scheffe test) indicated reliably lower levels of p-cofilin to total cofilin ratio in unimpaired mice relative to either memory impaired mice (*p* < 0.01) or unimpaired mice infused with proBDNF (*p* < 0.01), but no significant difference in p-cofilin levels between memory impaired mice and unimpaired mice infused with proBDNF (Figure 4A).

## Discussion

The current study evaluated the role of proBDNF and of the p75NTR neurotrophin receptor in aging-associated learning and memory deficits. Our results revealed that proBDNF was increased in the aged mouse hippocampus, compared to young, possibly as a result of decreased tPA and plasmin activation; proBDNF levels negatively correlated with good performance (RM and WM) in a water radial maze task. Infusions of “uncleavable” proBDNF into the CA1 region of the dorsal hippocampus significantly impaired memory recall in mice that previously learned the task, while blocking p75NTR association with RhoGDI using the TAT-Pep5 peptide improved performance in memory-impaired aged mice. These effects were gradual (over daily sessions) rather than immediate; this suggests that TAT-Pep5 affected not solely memory recall but also learning.

Changes in both neurotrophins and neurotrophin receptor levels occur during aging and neurodegeneration. In aged rats, proBDNF levels are elevated in the hippocampus while proNGF is elevated both in the hippocampus and cortex (Perovic et al., 2013). Increased proBDNF levels were also found in human and mouse Down’s syndrome brains, a disorder characterized by learning and memory deficits and neuronal degeneration similar to AD (Iulita et al., 2014). Our study revealed a similar elevation of proBDNF levels in the aged mouse hippocampus. A reduction in mature BDNF was reported in the aged human parietal and frontal cortices (Ferrer et al., 1999), hippocampus (Phillips et al., 1991; Hock et al., 2000) and nucleus basalis (Fahnestock et al., 2002), and in cortex in mouse models of AD (Peng et al., 2009), however, our study did not reveal significant changes in mature BDNF levels in the aged mouse hippocampus, but only increases in proBDNF. As previously observed in the hippocampus in aged humans (Webster et al., 2006) and rats (Silhol et al., 2005), our study indicated a reduction in trkB receptor levels in aged mice. Moreover, in similarity to studies reporting that p75NTR is increased in the aged brain (Costantini et al., 2006) but lost with AD progression (Mufson et al., 2002), we have found an increase in p75NTR in the aged mouse hippocampus.

Interestingly, studies using heterozygous BDNF knock out (BDNF+/−) mice, which exhibit an approximately 50% reduction of BDNF protein levels compared to wild type littermates (Endres and Lessmann, 2012; Meis et al., 2012; Psotta et al., 2013), reveal seemingly discrepant findings for a BDNF requirement in hippocampus-dependent learning: Two studies observed slight impairments during training trials in 3–4 and 12 months old BDNF+/− mice (Uutela et al., 2012; Rantamäki et al., 2013), while a more recent study (Petzold et al., 2015) found age-dependent learning deficits in BDNF+/− mice starting at seven months of age and a positive correlation between individual learning performance and hippocampal BDNF protein levels of well-performing animals. Moreover, several studies using region-specific, inducible BDNF knock out mouse models reported deficits in spatial learning in hippocampus or forebrain-restricted BDNF knockout mice (Gorski et al., 2003; Heldt et al., 2007). Although these studies point to an important role of BDNF in hippocampal learning and memory, since the genetically-modified animals have chronically (life-long) reduced BDNF levels, it is unclear whether the deficits in learning found in these animals are linked directly to the BDNF deficit or to subsequent changes in multiple gene expression levels.

Preservation of neuronal numbers and electrophysiological properties with age, in humans and rodents (Barnes, 1979; West, 1993; Amrein et al., 2004), suggests that age-related memory deficits are not due to cell loss but to alterations of functional and anatomical connectivity within the hippocampus, or between the hippocampus and entorhinal cortex (Lister and Barnes, 2009). Stereological counts of synapses in aged relative to young rats revealed a 24% reduction in synapses in the dentate gyrus (Geinisman et al., 1992), consistent with electrophysiology (Barnes and McNaughton, 1980; Foster et al., 1991; Patrylo and Williamson, 2007). Alterations of the postsynaptic densities at Schaffer collateral synapses in stratum radiatum and reduced densities of dendritic spines on CA1 basal dendrites were found in aged memory-impaired rats (Nicholson et al., 2004) and mice (von Bohlen und Halbach et al., 2006).

Neuronal plasticity is an important process for learning and memory. Dendritic spines, the main target of excitatory input on pyramidal neurons in the hippocampus and cortex, are highly dynamic actin-rich structures (Landis and Reese, 1983; Matus, 2000). Spine dynamics has been suggested as a mechanism for memory formation or elimination (Matsuzaki, 2007; Kasai et al., 2010). Rapid reorganization of the actin skeleton is an important factor, and recent evidence suggests that the link between synaptic activity, spine density and morphology and synapse formation and maintenance is provided by signaling pathways converging on the actin cytoskeleton (Hotulainen and Hoogenraad, 2010); major players in these pathways are members of the family of small Rho GTPases (Rac, cdc42 and RhoA; Ethell and Pasquale, 2005; Tada and Sheng, 2006). Rho GTPases act as molecular switches, existing in an active GTP-bound and an inactive guanosine diphosphate (GDP)-bound state (Van Aelst and D’Souza-Schorey, 1997; Hall, 1998). The activation of Rho GTPases is mediated by guanine-nucleotide exchange factors (Rho-GEFs), and is followed by recruitment of several downstream effectors. Guanine nucleotide dissociation inhibitors (GDIs), negatively regulate Rho GTPases by sequestering Rho proteins and interfering with both the GDP/GTP exchange as well as with the GTP hydrolysis (Van Aelst and D’Souza-Schorey, 1997). In neurons, Rho GTPases are found in dendritic spines and are major hotspots in actin cytoskeleton regulation: RhoA activation is necessary for expression of LTP, via cofilin phosphorylation and inactivation (Rex et al., 2009); Rac and cdc42 regulate spine head formation, mainly by activating Arp2/3 complex-induced actin nucleation and inhibiting actin depolymerization (Irie and Yamaguchi, 2002; Wegner et al., 2008; Hotulainen et al., 2009). Mutations in several Rho GEFS and GAPs have been shown to cause mental retardation, schizophrenia or autism (Newey et al., 2005). For example, DISC1 (Disrupted in Schizophrenia) regulates dendritic spine morphology via Rac1 (Hayashi-Takagi et al., 2010) and mutations in the cofilin kinase PAK3 lead to X-linked mental retardation and memory impairments (Allen et al., 1998). Interestingly, LIMK-1 knockout mice, which are unable to regulate cofilin activity through phosphorylation, have enhanced hippocampal LTP (Meng et al., 2002). Loss of cofilin-mediated synaptic actin dynamics leads to impairment of all types of associative learning (Rust et al., 2010).

**Figure 5:**
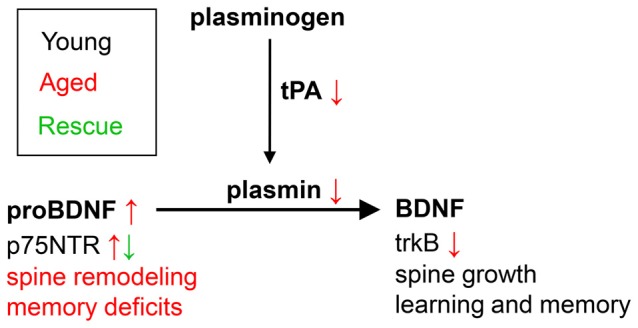
Theoretical model of the role of proBDNF in learning in memory in aged individuals. In young individuals, maturation of proBDNF to BDNF is controlled by plasmin and tPA, and is essential for learning and memory. Aged individuals show decreased levels of tPA and plasmin, and increased levels of uncleaved proBDNF, associated with increased spine remodeling and memory deficits. Blockade of p75NTR (e.g., by TAT-Pep5, as in the current study) leads to spine growth, and rescues learning and memory.

Signaling through the mature BDNF receptor, trkB, and the proneurotrophin receptor p75NTR have differential effects on the activation of neuronal Rho GTPases and spine morphology: trkB activation induces activation of the Rac1-Pak1 pathway, with actin polymerization and inhibition of cofilin-dependent actin depolymerization (Huang and Reichardt, 2003), inducing an increase in spine head size, while p75NTR was shown to associate with RhoGDI and modulate RhoA activity (Yamashita and Tohyama, 2003), inducing a reduction in spine density. ProBDNF reduces the density of dendritic spines in culture and this effect may be in part mediated by the p75NTR receptor via RhoA (Koshimizu et al., 2009). As an end target of RhoA, cofilin seems to be a particularly intriguing molecule: it can be found in different states (active/inactive, dephosphorylated/phosphorylated) and participates in many processes related to synaptic plasticity (Shaw and Bamburg, 2017). While many studies report that cofilin phosphorylation and actin polymerization are essential during the early phases of LTP (Chen et al., 2007; Zhang et al., 2010), constitutive cofilin activity is required to maintain spine head volumes, possibly through actin severing and increases in free barbed-ends, followed by actin polymerization to generate branched filaments and actin networks (Calabrese et al., 2014). Moreover, increased cofilin activity induces AMPA recruitment to the membrane after chemically-induced LTP (Gu et al., 2010). Thus our results showing an increase in p-cofilin in memory-impaired animals and after hippocampal proBDNF infusion on spatial memory in the WRAM task are particularly interesting. Modulation of cofilin activation and the effects on spine morphology and AMPA receptor trafficking, besides the role in promoting hippocampal LTD (Woo et al., 2005), could explain the effects of hippocampal proBDNF infusion on spatial memory observed in our study, supporting a theoretical model such as that presented in Figure 5.

Although the effects of proBDNF infusions on spatial memory are largely explained by the interaction with p75NTRs, since p75NTR is a common proneurotrophin receptor, the effects of TAT-Pep5 infusions may also reflect modulation of signaling pathways downstream proNGF or other proneurotrophins. Indeed, we have reported previously (Fortress et al., 2011) that a single intra-hippocampal proNGF injection in aged rats induces morphological changes in basal forebrain cholinergic neurons, with dendritic retraction and atrophy of cell bodies. Recent studies also showed that a reduction of p75NTR expression ameliorates the cognitive deficits (Murphy et al., 2015) and increases cholinergic innervation in the dentate gyrus (Dokter et al., 2015), basolateral amygdala (Busch et al., 2017) and visual cortex (Von Bohlen und Halbach and Von Bohlen und Halbach, 2016); moreover, a small molecule ligand of p75NTR (LM11A-31) reverses cholinergic neurite dystrophy in Alzheimer’s Disease mouse models (Simmons et al., 2014). Therefore, we cannot exclude that the improvement of spatial learning and memory in TAT-Pep5 infused mice reflects positive effects on basal forebrain cholinergic neurons and their hippocampal projections.

In summary, our study supports a role for the elevated p75NTR and proBDNF levels in the aged hippocampus in learning and memory deficits and supports p75NTR as a therapeutic target for aging-related memory impairments. Further studies are required to evaluate whether alterations in proBDNF levels occur in other brain regions relevant to spatial learning and memory. Also, since our study examined only aged male mice and given the important relationship between BDNF and estrogen (Luine and Frankfurt, 2013), additional experiments need to clarify whether similar changes occur in aged females.

## Author Contributions

MB: experimental design, behavior. MB and CVB: neuropharmacology, data analysis. MB and CE: biochemistry. MB, A-CG and CVB: manuscript preparation.

## Conflict of Interest Statement

The authors declare that the research was conducted in the absence of any commercial or financial relationships that could be construed as a potential conflict of interest.
